# The audio-visual integration effect on music emotion: Behavioral and physiological evidence

**DOI:** 10.1371/journal.pone.0217040

**Published:** 2019-05-30

**Authors:** Fada Pan, Li Zhang, Yuhong Ou, Xinni Zhang

**Affiliations:** School of Education Science, Nantong University, Nantong, China; University of Zurich, SWITZERLAND

## Abstract

Previous research has indicated that, compared to audio-only presentation, audio-visual congruent presentation can lead to a more intense emotional response. In the present study, we investigated the audio-visual integration effect on emotions elicited by positive or negative music and the role of visual information presentation durations. The participants were presented with audio-only condition, audio-visual congruent condition, and audio-visual incongruent condition and then required to judge the intensity of emotional experience elicited by the music. Their emotional responses to the music were measured using self-ratings and physiological aspects, including heart rate, skin temperature, EMG root mean square and prefrontal EEG. Relative to the audio-only presentation, the audio-visual congruent presentation led to a more intense emotional response. More importantly, the audio-visual integration occurred both in the positive music and in the negative music. Furthermore, the audio-visual integration effect was larger for positive music than for negative music; meanwhile the audio-visual integration effect was strongest with the visual information presented within 80s for negative music, which indicated that this integration effect was more likely to occur in the negative music. These results suggest that when the music was positive, the effect of audio-visual integration was greater. When the music was negative, the modulation effect of the presentation durations of visual information on the music-induced emotion was more significant.

## Introduction

In our daily life, we perceive the external world by processing information from multiple sensory modalities involving vision, hearing, touch, and so on. For example, when we hold a cup in our hands, both vision and touch provide meaningful information about the shape of the cup. This phenomenon is known as multisensory integration, whereby stimuli from multiple sensory modalities interact to form a coherent and meaningful representation [1]. The McGurk effect demonstrated the multisensory integration effect (i.e., audio-visual integration). McGurk and MacDonald found that when a film of a young woman repeating the syllable [ba] was dubbed on to the lip movements as [ga], individuals also reported hearing [da] [2]. After this study, many scholars started to examine the neural mechanism for audio-visual integration. Calvert et al. found that the audio-visual integration effects started at the early stage [3]. Exploring this problem further, Calvert et al. investigated the neural mechanism for audio-visual integration again and identified an area of the heteromodal cortex in the left superior temporal sulcus that exhibited significant supra-additive response enhancement to match audio-visual inputs [4]. In recent years, more and more electrophysiological studies have shown that audio-visual integration occurred at multiple neural areas, such as the superior temporal gyrus [5, 6], the left inferior frontal gyrus, the left somatosensory association cortex, and the left supramarginal cortex [7].

A large number of studies showed that the information, including emotional information, from different sensory modalities could be integrated. The McGurk effect was also suitable for the study of audio-visual integration in emotion. A study by de Gelder explored the combination of emotional information from facial expression and voice tone [8]. The results showed that the judgment of facial emotion was affected by the auditory information and that the effective integration would occur even though the emotional information from visual and auditory modalities was conflicted. The same effect was more obvious in the judgment of fear. When accompanied by a fearful voice, the subjective self-rating expressed more fear whether the visual stimulus was fearful or neutral. However, no such influence on the rating of faces was found for happy voices [9].

Research over the past two decades demonstrated that numerous cortical and subcortical brain regions, such as the superior colliculus (SC), the superior temporal sulcus (STS), and the parietal, premotor and prefrontal cortices, were involved in audio-visual integration [10]. However, at least two questions remained unanswered: 1) What was the neural mechanism for audio-visual integration in emotion? and 2) At which stage of cognitive processing did integration take place, and which brain structure was related to it? A large number of studies showed that the integrated area of emotional face-sound was related to the middle temporal gyrus (MTG) [9, 11, 12], the posterior superior temporal sulcus (pSTS) [9], and the posterior superior temporal gyrus (pSTG) [13]. Moreover, compared with stimuli from the single modality, audio-visual stimuli also activated the thalamus [13]. Furthermore, the activation for fear-face and fear-voice was the amygdala [14], whereas the activation for happy-face and happy-voice was the hippocampus, the claustrum, and the inferior parietal lobule. In sum, the integration of different emotional information activate common as well as specific brain regions [11].

Further studies showed that audio-visual integration has certain time properties. According to the experimental results, the integration of auditory and visual information was extremely fast and automated, and the interaction of visual and auditory information was likely to occur at an early stage [15, 16]. Frassinetti et al. found that the effect of audio-visual integration was enhanced only when stimuli in the two different modalities were presented synchronously [15]. In other words, the visual and auditory stimuli must be presented at the same time for the enhancement of audio-visual integration effect to occur. However, previous research suggested that it was not necessary to present the visual and auditory information synchronously to induce the audio-visual integration effect. This effect could also appeared when the stimuli from two sensory modalities subsequently input in a short interval [16, 17]. Moreover, the time window, which was typically between 150 and 450ms, depended on the experimental material and the experimental paradigm [16]. The latest study used BOLD-FMRI to research the neural substrates involved in processing temporal synchrony and asynchrony with audio-visual signals. The results showed that S-mSTC (Synchrony-defined multisensory superior temporal cortex) responded only to synchronous audiovisual stimulus presentations, with no activation observed at asynchrony levels greater than or equal to 100ms, while B-mSTC showed activation with any multisensory audiovisual speech stimulus and increased activation as stimulus asynchrony increased [18]. These results showed that the audio-visual integration effects were diverse at different time windows, which activated specific brain regions.

At the same time, the audio-visual integration of emotional information also occurred at the early stage of processing. Pourtois et al. suggested that the combination of the two sensory modalities occurred in a short time window (110ms) and translated as a specific enhancement in the N1 component [19]. Another study found that the combination of the audio-visual sensory modalities translated as a reduction in N1, P2, and N3 components [20, 21]. In other words, the audio-visual integration occurred at about 100ms. Similarly, Chen et al. pointed out that N1 and P3 amplitudes were larger for the bimodal-change conditions [22]. The results suggested that the facial-vocal integration during emotional change perception was subserved by at least two processes denoted by N1 and P3 [22] and that the time of audio-visual integration was about 300ms. Overall, the audio-visual integration happened within 300ms, but the time window was affected by the task requirements, such that the integration might take longer (i.e., over 300ms) if different tasks were employed.

Research over the past two decades has shown that the perception of musical emotion could be influenced by visual cues, such as body postures, gestures, and facial expressions [22, 23]. When the music and visual information were presented synchronously, it was unclear whether the emotional information from the two sensory modalities would be potentially integrated or not. Some studies demonstrated that the audio-visual integration would occur in the musical emotion expression, while other studies indicated that the two sensory modalities would not be integrated. Vines et al. [24] and Vuoskoski et al. [25] found that AO (audio-only presentation) elicted the strongest emotional reaction in the galvanic skin response and that there was no significant difference between AV (audio-visual presentation) and AO. These results indicated that audio-visual presentation did not induce a higher emotional response than unimodal (i.e., visual-only or audio-only) presentation. In other words, musical and visual information would not be integrated. However, additional research offered different results. Chapados and Levitin discovered that there was a significant effect between AV and AO, and between AV and VO (visual-only presentation) in the galvanic skin response. This suggested that the interaction effect between the multiple sensory modalities (audio and visual sensory modalities) was larger than the effect caused by the single sensory modality in the expression of music communication [23]. Moreover, Platz conducted a meta-analysis to examine the emotional expression elicited by music and supported the result that audio-visual presentations enhanced musical appreciation [26]. In other words, music and visual information were integrated. Some studies also discovered the integration effect even when musical emotion and visual emotion were different. Weijkamp and Sadakata pointed out that there was an interference effect when the stimuli from the visual sensory modality and the auditory sensory modality were incongruent [27]. Recently, researchers using FMRI technology analyzed this phenomenon. Jeong et al. observed that congruence and incongruence of auditory and visual stimuli might be integrated through increments and decrements in neuronal activity in the superior temporal gyrus (STG) and the fusiform gyrus (FG). There was increased activation in the STG under the congruent condition. However, there was decreased activation in the STG and increased activation in the FG under the incongruent condition [28]. To summarize, most research had verified that music-induced emotion was influenced by visual stimulation. However, future research is required to explain whether the two sensory modalities inputs would be integrated.

It should be mentioned that there are two limitations in the previous studies regarding the audio-visual integration effect on the emotion elicited by music. First, there is no separation between positive and negative emotions when studying whether visual information can influence music-induced emotion. Some studies on multisensory processing have indicated that all bimodal conditions could induce significantly strong activation in the superior temporal gyrus (STS), the inferior frontal gyrus (IFG), and the parahippocampal gyrus, including the amygdala. By contrast, some studies suggested that different emotions had different mechanisms of emotional processing. For instance, the structures activated by happy audio-visual pairs were mainly lateralized in the left hemisphere, whereas the structures activated by fearful audio-visual pairs were lateralized in the right hemisphere [12]. Above all, emotional activation regions imply that all types of emotional information not only have a common neural basis but also have unique integration characteristics. Therefore, the different types of emotion should be separately processed when we explore the audio-visual integration effects on emotions. Second, according to previous studies, we found that the audio-visual integration effect on both cognitive and emotional processing had certain time properties. Previous studies have indicated that when the two sensory modalities were presented at the same time, or the time window was at 300ms, the information from the two sensory modalities would be integrated. This is also verified in musical emotion. Thus, further research is needed to determine whether a stronger integration effect occurs when the presentation durations of visual information are longer.

In brief, the present study aims to investigate the audio-visual consistency effects in emotions elicited by positive and negative music. Furthermore, we will examine whether presentation durations of visual information can modulate the audio-visual integration effects elicited by music. In general, we hypothesize that the congruence of auditory and visual emotions will promote the inducing of the emotion. Additionally, we expect that the presentation durations of visual information will modulate the effect of music-induced emotion for audio-visual integration.

## Experiment 1

“This study was approved by the Human Ethics Committee of Nantong University. A written informed consent was obtained from all participants, each of whom was given a small gift in return for their participation.”

### Methods

#### Participants

Thirty-two healthy undergraduate students (16 males and 16 females ranging from 17 to 26 years of age, with an average age of 20.75 years) volunteered to participate in the present study. All participants were right-handed, had no mental illness, color blindness or color weakness, and had normal or corrected-to-normal vision.

#### Stimulus material

**Visual stimuli.** The pictures in the present study were selected from the Chinese Facial Affective Picture System [29]. We obtained the ratings of the Arousal and Valence from this database (Chinese Facial Affective Picture System) and then tested the significance of differences between the positive and negative pictures. We obtained the ratings of the Arousal and Valence from this database (Chinese Facial Affective Picture System) on a 9-point scale and then tested the significance of differences between the positive and negative pictures. According to t-test analysis, the average valence ratings between the positive (i.e., happy) pictures (*M* = 7.44) and the negative (i.e., sad) pictures (*M* = 2.20) were significantly different (*t* = 12.65, *p* < 0.01). In addition, the average arousal ratings of positive (i.e., happy) and negative (i.e., sad) pictures were 7.16 and 6.67, respectively. The result of the t-test showed no significant difference on the average arousal ratings between these two groups of pictures (*t* = 0.82, *p* > 0.05). When the participants did the audio-only task, the visual stimuli was a 35% gray level picture. All pictures were presented in jpg format, at a resolution of 260 × 300 pixels. These visual stimuli were presented for 120 seconds at the center of a 35% gray level computer background.

**Auditory stimuli.** Four pieces of music were selected, including two positive (i.e., happy) music clips and two negative (i.e., sad) music clips. The happy music was Piano Concerto No. 23 in A Major, K. 488-(2), by Wolfgang Amadeus Mozart, and Brandenburg concerto No. 2, by Johann Sebastian Bach. The sad music was Adagio, by Tomaso Albinoni, and Kol Nidrei, by Max Bruch. Before the formal experiment, 15 participants were randomly selected to evaluate the music pieces. The valence and arousal were scored with a 9-point scale for music pieces. According to the t-test analysis, the average valence ratings of positive (i.e., happy) and negative (i.e., sad) music clips were 6.43 and 2.80, respectively. The result of the t-test showed that the difference between them was significant (*t* = 9.19, *p* < 0.001). However, there was no significant difference on the average arousal ratings between the positive (i.e., happy) music clips (*M* = 6.83) and the negative (i.e., sad) music clips (*M* = 6.80) (*t* = -0.08, *p* > 0.05). All the music segments were presented for 120 seconds in MP4 format.

**Subjective self-rating scale.** On the 9-point self-rating scale for the degree of happiness, “1” represented “not happy at all,” “5” represented the middle degree of happiness, and “9” represented “very happy.” This is similar to the self-rating for the degree of sadness, where “1” represented “not sad at all,” “5” represented the middle degree of sadness, and “9” represented “very sad.”

#### Procedure

In Experiment 1, a 2 (music emotion type: positive emotion, negative emotion) × 3 (audio-visual congruence: audio-visual congruent, audio-visual incongruent, control group) ×2 (valence: positive, negative) factorial design was applied. Valence is the extent to which an individual is made happy or sad by the stimuli. In this present, it included happiness and sadness. The experimental stimuli consisted of audio-visual congruence (AV-C), audio-visual incongruence (AV-I) and control group (audio-only: AO) versions. For the stimuli of audio-visual congruence, the audio and visual emotions were congruent (e.g., happy face and happy music). Specifically, when the happy music were presented, the participants would see a happy face at the center of 35% gray level computer background. For the stimuli of audio-visual incongruence, the audio and visual emotions were incongruent (e.g., happy face and sad music). Specifically, when the sad music were presented, the participants would see a happy face at the center of 35% gray level computer background. For the stimuli of control group (audio-only), when the music were presented, the participants would see a 35% gray level computer background. The duration of each stimulus was 120 seconds. The experimental procedure and stimulus presentation were edited with PowerPoint 2016.

Before the formal experiment, participants were required to sit and rest for 5 minutes in front of the computer screen. They were then informed about the experimental tasks and signed the consent form. Then, the physiological sensors were connected to the participant’s non-dominant hand. When all signal indicators were stable, asking the participants to press the “start” button to start the experiment. In the experiment, music was played through the “Mi” noise-canceling headphones (model: type-C), while it was played with emotional face or grey picture simultaneously. Then, the participants judged the emotion induced by music after playing the music, and scored 1–9 grades for the the happiness ratings and sadness ratings (lasting 60 seconds). At the end of judgment, the participants were asked to complete 10 arithmetic questions (lasting 60 seconds), which enabled them to return to a calm, natural state and prepare for the next stimulus.

To balance the differences of the stimuli and reduce practice effects, we set up that each piece of music corresponds to a happy facial picture, a sad facial picture and a 35% gray picture. Twelve trials were presented in the study, and the length of the study was approximately 48 minutes.

#### Bio-signal recording

The equipment used was an MP150 (Biopac System, U.S) to measure all ECG (Electrocardiography), EMG (Electromyography), SKT, and EEG (electroencephalogram) signals. For ECG signals, the amplifier gain was set at 500, the high-pass filter was set at 0.5 Hz, and the low-pass filter was set at 35 Hz. Two electrodes were attached to the heart on the up and down. The white lead was slightly upward on the sword process. The red lead was attached to the left 3–4 ribs. The black lead was the ground pole and attached on the right side of the navel. For EMG signals, the amplifier was set at 2000, the high-pass filter was set at 1 Hz, and the low-pass filter was at 100 Hz HPN OFF. Two electrodes were attached to the left hand to collect electromyography. For SKT, the amplifier was set at 1000, the high-pass filter was set at DC, and the low-pass filter was at 10 Hz. The skin temperature sensor was attached behind the neck. For EEG signals, the amplifier was set at 5000, the high-pass filter was set at 0.5 Hz, and the low-pass filter was at 35 Hz. Two electrodes were attached at the frontal area.

#### Data analysis

For ECG (Electrocardiography), EMG (Electromyography) and SKT, we chose the median of one minute in each musical segment to test the difference. For the electroencephalogram (EEG), we filtered the frequency of each type of brain wave, then copied the waves of the median of 1 minute in each musical segment. Then we took the waves to Fast Fourier Transform (FFT) to obtain the maximum power. Repeated-measures ANOVAs were conducted in SPSS 22.0.

### Results

#### Subjective self-ratings

A 2 (music type: positive music, negative music) ×3 (audio-visual congruence: audio-visual congruent, audio-visual incongruent, control group) ×2 (valence: positive, negative) repeated-measures ANOVA was performed on the subjective self-ratings. A significant effect of music type was observed, *F*(1,31) = 4.78, *p* < 0.05, *η*^*2*^ = 0.14. The subjective self-ratings for positive music were significantly lower than those for negative music. The main effect of valence was not significant, *F*(1,31) = 0.18, *p* > 0.05. The main effect of audio-visual was also not significant, *F*(2,62) = 2.83, *p* > 0.05.

The three-way interaction of music type, audio-visual congruence and valence was significant, *F*(2, 62) = 38.67, *p* < 0.001, *η*^*2*^ = 0.56. After studying the interaction further, the interaction between music type and audio-visual congruence was significant in the positive valence, *F*(2, 62) = 22.35, *p* < 0.001, *η*^*2*^ = 0.42. Further examination of the interaction between music type and audio-visual congruence revealed a significant difference between audio-visual congruence when the music was negative, *F*(2, 62) = 7.02, *p* < 0.01, *η*^*2*^ = 0.32. Specifically, the self-ratings of the condition of audio-visual incongruence and control group were higher than the condition of audio-visual congruence. Meanwhile, it showed an even larger effect of audio-visual congruence for positive music, *F*(2, 62) = 19.43, *p* < 0.001, *η*^*2*^ = 0.56. Specifically, the self-ratings of the condition of audio-visual incongruence and audio-visual congruence were higher than the condition of control group.

Meanwhile, the interaction between music type and audio-visual congruence was significant in the negative valence, *F*(2, 62) = 35.04, *p* < 0.001, *η*^*2*^ = 0.53. Further examination of the interaction between music type and audio-visual congruence revealed a significant difference between audio-visual congruence when the music was negative, *F*(2, 62) = 12.34, *p* < 0.001, *η*^*2*^ = 0.45. Specifically, the self-ratings of the condition of audio-visual congruence and incongruence were lower than the condition of control group. Meanwhile, it showed an even larger effect of audio-visual congruence for positive music, *F*(2, 62) = 29.22, *p* < 0.001, *η*^*2*^ = 0.66. Specifically, the self-ratings of the condition of audio-visual incongruence and control group were lower than the condition of audio-visual congruence.

**Fig 1:**
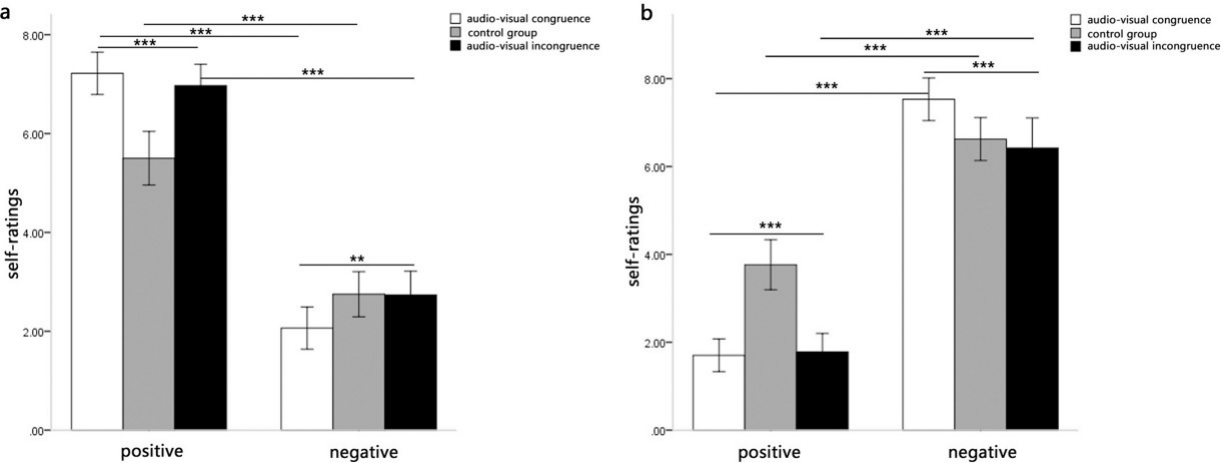
Means self-ratings of music-induced emotion as a function of music type and audio-visual congruence. Fig 1A displays the results of Repeated measures analysis in the self-rating scores of happiness. Fig 1B displays the results of Repeated measures analysis in the self-rating scores of sadness.

These results revealed that the positive music induced significant positive emotion and the negative music induced significant negative emotion. Moreover, this indicated that the difference of audio-visual congruence was significant in both positive and negative music conditions, and the difference among the three conditions (audio-visual congruence, audio-visual incongruence and audio only) was larger in positive music than that in negative music. More importantly, the strength of individuals’ emotional experienced intensity when the facial emotion and musical emotion were congruent. However, when the music was positive, the negative facial emotion could also enhance individuals’ emotional experienced intensity, which the positive facial emotion didn’t have significant effect when the music was negative (Fig 1).

#### Heart rate

A 2 (music type: positive emotion, negative emotion) × 3 (audio-visual congruence: audio-visual congruent, audio-visual incongruent, control group) repeated-measures ANOVA was performed on the heart rates. A significant effect of audio-visual congruence was observed, *F*(2,62) = 5.01, *p* < 0.05, *η*^*2*^ = 0.14. The heart rates for the audio-visual congruent condition were significantly lower than those for the control group, while the heart rates for the audio-visual incongruent condition were significantly higher than those for the control group. This suggested that the visual emotional information had a significant effect on music-induced emotion. That means, when the valence of visual stimuli was congruent with the musical stimuli, it would decrease the heart rates and enhance the emotional experience. Oppositely, it would enhance the heart rates and decrease the experience. However, the main effect of music type was not significant, *F*(1, 31) = 3.29, *p* > 0.05.

**Fig 2:**
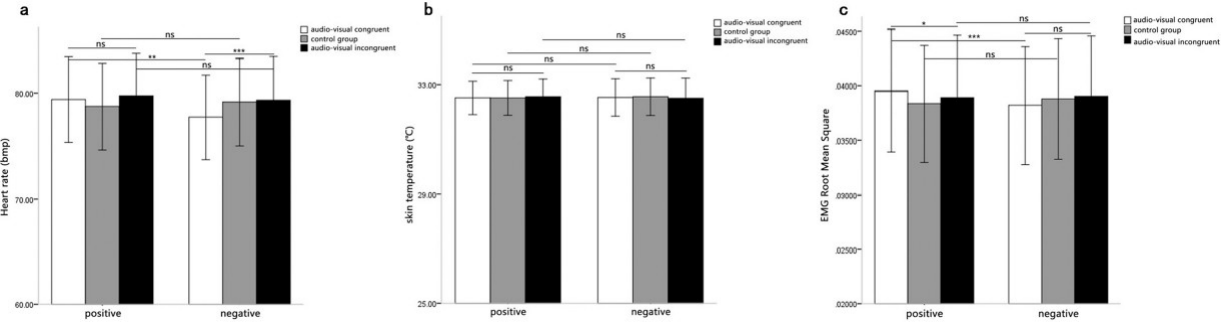
Means physiological indicators of music-induced emotion as a function of music type and audio-visual congruence. Fig 2A displays the results of Repeated measures analysis in heart rates. Fig 2B displays the results of Repeated measures analysis in skin temperatures. Fig 2C displays the results of Repeated measures analysis in EMG root mean squares.

The interaction between music type and audio-visual congruence was significant, *F*(2, 62) = 4.17, *p* < 0.05, *η*^*2*^ = 0.12. Simple-effect tests showed a significant difference among the three conditions (audio-visual congruence, audio-visual incongruence and audio only) when the music was negative, *F*(2, 62) = 14.33, *p* < 0.001, *η*^*2*^ = 0.49, with the heart rates lower for audio-visual congruence than for audio-visual incongruence. The audio-visual congruence effects were not significant for the positive music, *F*(2, 62) = 2.48, *p* > 0.05 (Fig 2A).

#### Skin temperature

The repeated measures ANOVAs on skin temperature showed that the main effect of music type was not significant, *F*(1, 31) = 0.001, *p* > 0.05, and the main effect of audio-visual congruence was also not significant, *F*(2, 62) = 0.69, *p* > 0.05. The interaction between music type and audio-visual congruence was not significant, *F*(2, 62) = 0.35, *p* > 0.05 (Fig 2B).

#### EMG root mean square

The repeated measures ANOVAs on EMG root mean square showed the main effect of music type was not significant, *F*(1, 31) = 2.60, *p* > 0.05, and the main effect of audio-visual congruence was also not significant, *F*(2, 62) = 0.96, *p* > 0.05. The interaction between music type and audio-visual congruence was significant, *F*(2, 62) = 6.24, *p* < 0.01, *η*^*2*^ = 0.17. Simple effect analysis showed that the audio-visual congruence effects were only significant for the positive music, *F*(2, 62) = 3.89, *p* < 0.05, *η*^*2*^ = 0.21, with the root mean square larger for audio-visual congruence than for incongruence. The audio-visual congruence effects were not significant for the negative music, *F*(2, 62) = 2.96, *p* > 0.05 (Fig 2C).

#### Prefrontal EEG

**Fig 3:**
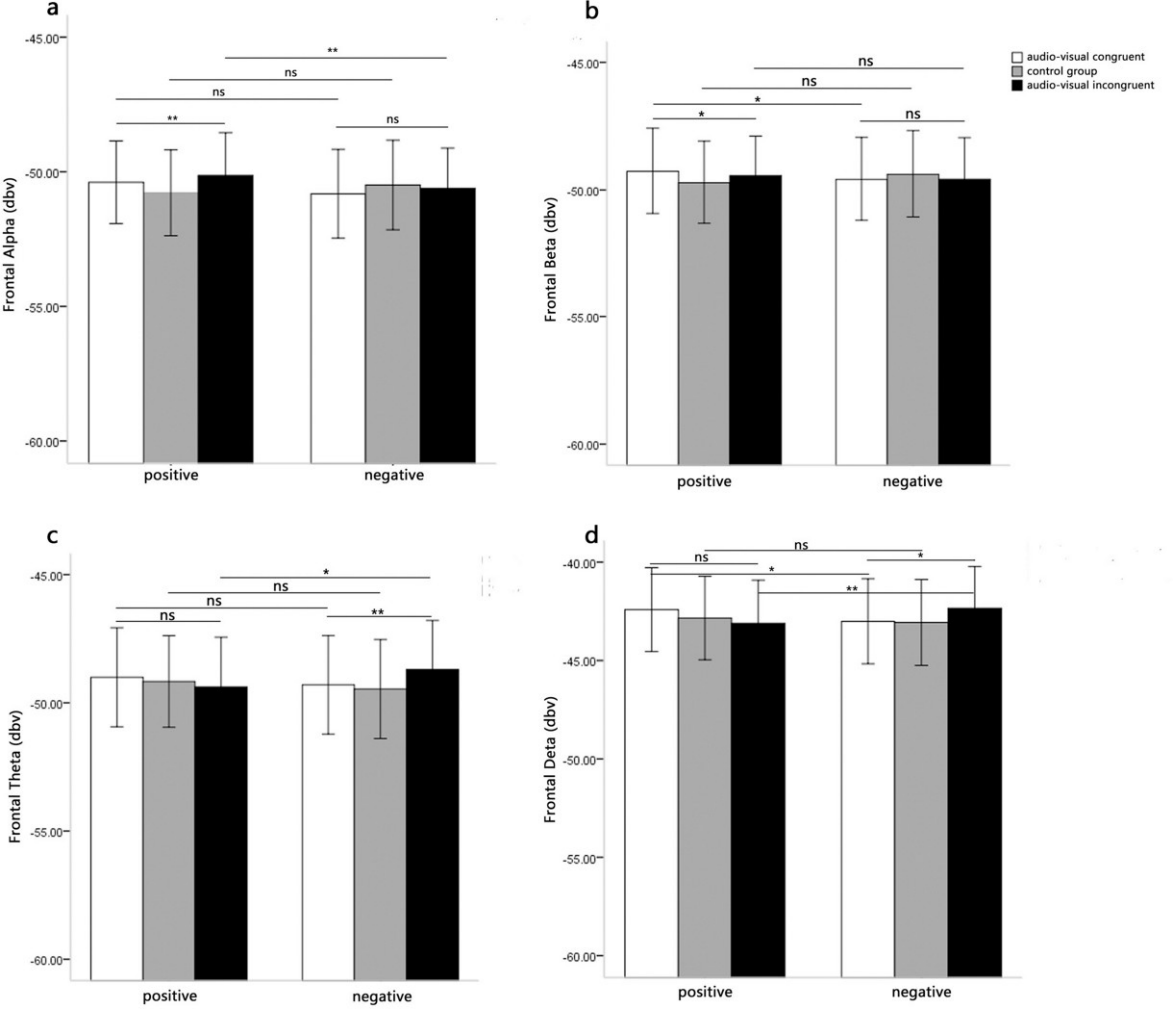
Means EEG power of music-induced emotion as a function of music type and audio-visual congruence. Fig 3A displays the results of Repeated measures analysis in prefrontal alpha power. Fig 3B displays the results of Repeated measures analysis in prefrontal beta power. Fig 3C displays the results of Repeated measures analysis in prefrontal theta power. Fig 3D displays the results of Repeated measures analysis in prefrontal delta power.

The analysis of alpha power showed a significant main effect of music type, *F*(1, 31) = 5.05, *p* < 0.05, *η*^*2*^ = 0.14, and a significant interaction between music type and audio-visual congruence, *F*(2, 62) = 4.49, *p* < 0.05, *η*^*2*^ = 0.13. However, the main effect of audio-visual congruence was not significant, *F*(2, 62) = 2.45, *p* > 0.05. Simple effect analysis showed the audio-visual congruence effects were only significant for the positive music, *F*(2, 62) = 9.08, *p* < 0.001, *η*^*2*^ = 0.38, with the alpha power lower for audio-visual congruence than for audio-visual incongruence. The audio-visual congruence effects were not significant for the negative music, *F*(2, 62) = 2.68, *p* > 0.05 (Fig 3A).

The repeated measures ANOVAs on beta power showed the main effect of music type was not significant, *F*(1, 31) = 0.22, *p* > 0.05, and the main effect of audio-visual congruence was also not significant, *F*(2, 62) = 0.88, *p* > 0.05. The interaction between music type and audio-visual congruence was significant, *F*(2, 62) = 4.81, *p* < 0.05, *η*^*2*^ = 0.13. Simple effect analysis showed that the audio-visual congruence effects were only significant for the positive music, *F*(2, 62) = 2.96, *p* = 0.06, *η*^*2*^ = 0.17, with the beta power larger for audio-visual congruence than for incongruence. The audio-visual congruence effects were not significant for the negative music, *F*(2, 62) = 1.45, *p* > 0.05 (Fig 3B).

The repeated measures ANOVAs on theta power showed the main effect of music type was not significant, *F*(1, 31) = 0.04, *p* > 0.05, and the main effect of audio-visual congruence was also not significant, *F*(2, 62) = 1.53, *p* > 0.05. The interaction between music type and audio-visual congruence was significant, *F*(2, 62) = 3.63, *p* < 0.05, *η*^*2*^ = 0.11. Simple effect analysis showed that the audio-visual congruence effects were only significant for the negative music, *F*(2, 62) = 6.01, *p* < 0.01, *η*^*2*^ = 0.29, with the theta power lower for audio-visual congruence than for incongruence. The audio-visual congruence effects were not significant for the positive music, *F*(2, 62) = 0.94, *p* > 0.05 (Fig 3C).

The repeated measures ANOVAs on delta power showed the main effect of music type was not significant, *F*(1, 31) = 0.02, *p* > 0.05, and the main effect of audio-visual congruence was also not significant, *F*(2, 62) = 0.98, *p* > 0.05. The interaction between music type and audio-visual congruence was significant, *F*(2, 62) = 5.31, *p* < 0.01, *η*^*2*^ = 0.15. Simple effect analysis showed that the audio-visual congruence effects were only significant for the negative music, *F*(2, 62) = 4.13, *p* < 0.05, *η*^*2*^ = 0.22, with the delta power lower for audio-visual congruence than for incongruence. The audio-visual congruence effects were not significant for the positive music, *F*(2, 62) = 2.16, *p* > 0.05 (Fig 3D).

### Discussion

Experiment 1 was designed to explore the effect of visual information on music-induced emotion in different emotional states. The results of Experiment 1 revealed that when presented with a visual stimulus (i.e., face) and an auditory stimulus (i.e., music) synchronously, participants began to combine the two sensory modalities of information. The combination was manifested in both the subjective responses and the physiological aspects. More concretely, the differences of audio-visual congruence were significant both in positive and negative emotional conditions, and there was a larger difference between the audio-visual congruent and incongruent conditions for positive music than for negative music in the subjective responses. Similar results were found in the alpha power, beta power of the prefrontal area, and the EMG root mean square, which means that the difference among audio-visual congruence, control group and audio-visual incongruence was significant when the auditory stimulus is positive rather than negative. However, we obtained the opposite results for heart rate where the difference the three conditions (audio-visual congruence, audio-visual incongruence and audio only) was significant when the auditory stimulus was negative rather than positive. Moreover, these results revealed that when the music and facial emotions were congruent, individuals would evoke more intense emotion. Except the theta power and delta power of prefrontal areas, the other indexes showed that when the music and facial emotions were incongruent, the visual information had no influence on the music-induced emotion.

On the one hand, these results revealed that the audio-visual integration effect occurred in the music-induced response. Additionally, the results were consistent with previous studies. For example, Chapados and Levitin found that the interaction effect of auditory and visual stimuli was greater than the effect of a single sensory modality in the musical performance [23]. Chen et al. also supported the view that bimodal emotional changes were detected with shorter response latencies compared to unimodal condition [22]. This suggested that bimodal emotional cues facilitated emotional change detection and that an integration effect of bimodal emotional cues occurred. On the other hand, we found that the audio-visual integration effect was larger for the positive music. According to the attention distribution theory, positive emotion can broaden an individual’s breadth of attention, while negative emotions tend to narrow an individual’s breadth of attention [30]. Thus, the positive music broadened the range of attention [31] and the participants were more likely to be attracted by the visual information. In other words, when the musical and facial emotions were both positive, enhancing the combination of visual and auditory sensory stimuli, it became easier for the participants to perceive the emotional information from the facial picture.

## Experiment 2

“This study was approved by the Human Ethics Committee of Nantong University. Written informed consent was obtained from all participants, each of whom was given a small gift in return for his or her participation.”

### Methods

#### Participants

Thirty-two healthy undergraduate students (17 males and 15 females ranging from 17 to 26 years of age, with an average age of 20.84 years) volunteered to participate in the present study. All participants were right-handed, had no mental illness, color blindness, or color weakness, and had normal or corrected-to-normal vision.

#### Stimulus material

**Visual stimuli.** The visual stimuli were six pictures selected from the Chinese Facial Affective Picture System [29], including three positive (i.e., happy) and three negative (i.e., sad) pictures. According to t-test analysis, a significant difference occurred on the average valence ratings between the positive (i.e., happy) pictures (*M* = 7.33) and the negative (i.e., sad) pictures (*M* = 2.37) (*t* = 24.71, *p* < 0.001). In addition, the average arousal ratings of positive (i.e., happy) and negative (i.e., sad) pictures were 6.55 and 6.33, respectively. The result of the t-test showed no significant difference on the average arousal ratings between these two groups of pictures (*t* = 0.38, *p* > 0.05). All pictures were presented in jpg format, at a resolution of 260 × 300 pixels.

**Auditory stimuli.** Six pieces of music were selected, including three positive (i.e., happy) musical clips and three negative (i.e., sad) musical clips. The happy music was Piano Concerto No. 23 in A Major, K. 488-(2), by Wolfgang Amadeus Mozart; Brandenburg concerto No. 2, by Johann Sebastian Bach; and Divertimento in D Major, K. 251—IV. Menuetto, by Mozart. The sad music was Adagio, by Tomaso Albinoni; Kol Nidrei, by Max Bruch, and Peer Gynt Suite (Aase’s Death), by Edvard Grieg. According to the t-test analysis, there was significant difference on the average valence ratings between the positive music (*M* = 6.53) and the negative music (*M* = 2.73) (*t* = 11.68, *p* < 0.001). Additionally, the average arousal ratings of positive and negative music were 6.80. The result of the t-test showed no significant difference on the average arousal ratings between these two groups of pictures (*t* = 0.52, *p* > 0.05). To ensure the homogeneity of the three pieces of positive music, the differences on the valence and arousal should be not significant. Therefore, the one–way repeated-measures ANOVA was used on the valence and the arousal. The results showed that the main effect of valence was not significant, *F*(2, 28) = 1.49, *p* > 0.05, *η*^*2*^ = 0.09 and the main effect of arousal was also not significant, *F*(2, 28) = 2.37, *p* > 0.05, *η*^*2*^ = 0.02. For the negative music, we did the same analysis. The results showed that the main effect of valence was not significant, *F*(2, 28) = 0.87, *p* > 0.05, *η*^*2*^ = 0.06; the main effect of arousal was also not significant, *F*(2, 28) = 0.95, *p* > 0.05, *η*^*2*^ = 0.06. All the music segments were presented for 120 seconds in MP4 format.

#### Procedure

**Fig 4:**
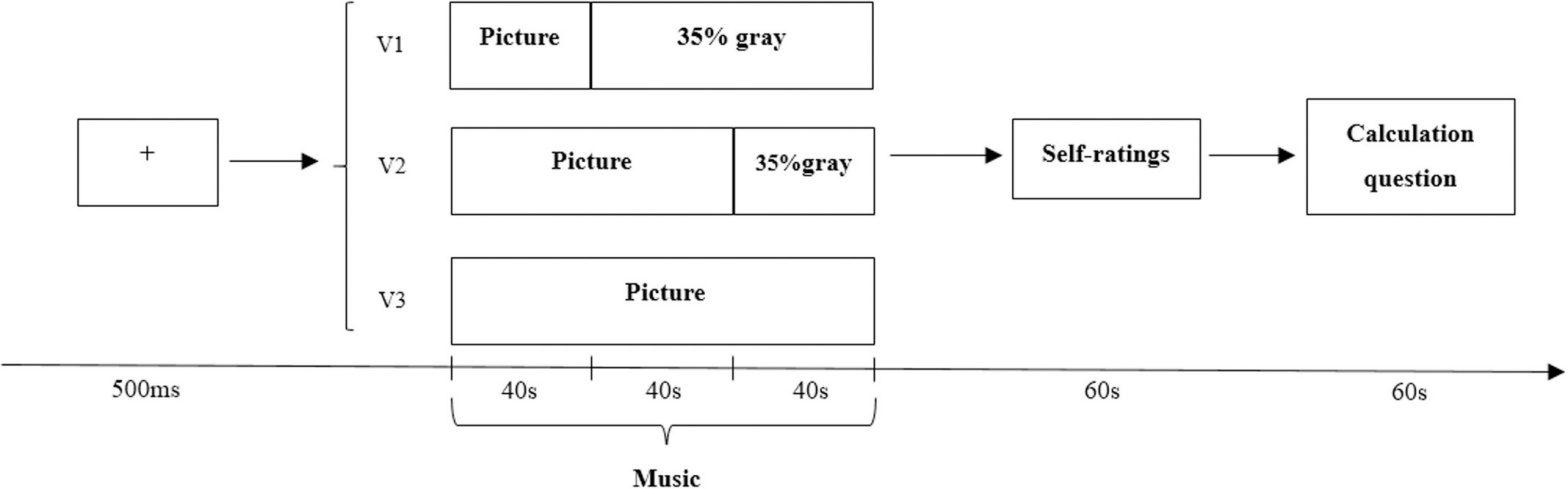
Scheme of a trial’s time course. All trials started with a fixation cross lasting for 500ms. Then, there were three conditions. The first one consisted in a picture shown for 40s and then a gary screen for 80s. In the second one, the picture was shown for 80s, followed by a gray screen for 40s, whereas in the third one the picture was shown for 120s. All conditions were accompanied by a piece of music (120s). At the end of each trial, participants had to judge the music-induced emotion and complete 10 arithmetic questions.

In Experiment 2, a 2 (music type: positive emotion, negative emotion) × 2 (audio-visual congruence: audio-visual congruent, audio-visual incongruent) × 3 (presentation durations: 40s, 80s, 120s) factorial design was employed that contained three blocks in total with each block containing four musical clips presented in a pseudorandom order. Half of the four trials were audio-visual congruent (i.e., happy music and happy facial pictures; sad music and sad facial pictures), and the other half were audio-visual incongruent (i.e., happy music and sad facial pictures; sad music and happy facial pictures). In Experiment 2, we set up three types of presentation durations of visual stimuli, which were 40s, 80s and 120s. The rest of Experiment 2 was the same as Experiment 1. The specific procedure was shown in Fig 4.

To balance the differences of the stimuli and reduce practice effects, we set up that each piece of music corresponds to a happy facial picture and a sad facial picture. Twelve trials were presented in the study, and the length of the study was approximately 48 minutes.

#### Data analysis

The results of experiment 1 showed that the interaction between music type and valence was significant. Specifically, the happiness ratings were significantly higher than the sadness ratings in the positive music and the result was just opposite in the negative music, which indicated that the positive music induced significant positive emotion and the negative music induced significant negative emotion. Based on thus results, we just chose the happiness ratings as the dependent variable for the happy music trials in experiment 2, and in the sad music trials, the sadness ratings were treated as the dependent variable.

### Results

#### Subjective self-ratings

A 2 (music type: positive emotion, negative emotion) × 2 (audio-visual congruence: audio-visual congruent, audio-visual incongruent) × 3 (presentation durations: 40s, 80s, 120s) repeated-measures ANOVA was performed on the subjective self-ratings. There was a significant difference of audio-visual congruence, *F*(1, 31) = 17.87, *p* < 0.001, *η*^*2*^ = 0.37, which indicated that the subjective self-ratings for the audio-visual congruent condition were significantly higher than those for the audio-visual incongruent condition. The main effect of presentation durations was also significant, *F*(2,62) = 3.84, *p* < 0.05, *η*^*2*^ = 0.11. Post hoc comparisons of means revealed that the self-ratings at 80s were significantly larger than those at 40s and 120s. The main effect of music type was not significant, *F*(1,31) = 1.81, *p* > 0.05.

**Fig 5:**
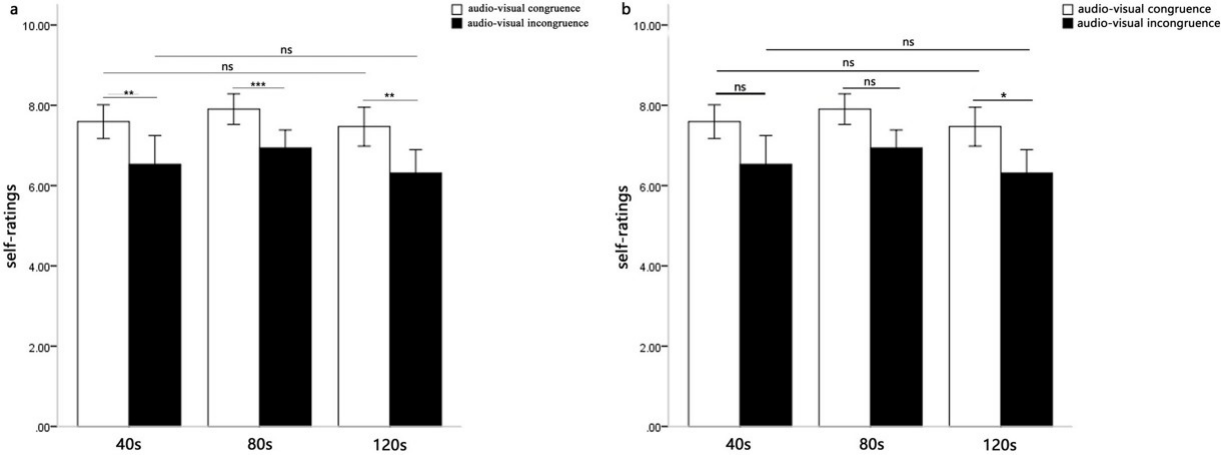
Means self-ratings of music-induced emotion as a function of audio-visual congruence and presentation durations. Fig 5A displays the results of Repeated measures analysis when the auditory stimuli (music) were positive. Fig 5B displays the results of Repeated measures analysis when the auditory stimuli (music) were negative.

However, the three-way interaction of audio-visual congruence, music type and presentation durations was not significant, *F*(2, 62) = 1.11, *p* > 0.05. Furthermore, the interaction between audio-visual congruence and presentation durations was not significant for the positive music, *F*(2, 62) = 0.15, *p*>0.05. The interaction between audio-visual congruence and presentation durations was not significant for the negative music, *F*(2, 62) = 1.02, *p*>0.05 (Fig 5).

#### Heart rate

A 2 (music type: positive emotion, negative emotion) × 2 (audio-visual congruence: audio-visual congruent, audio-visual incongruent) × 3 (presentation durations: 40s, 80s, 120s) repeated-measures ANOVA was performed on the heart rates. The main effect of music type was not significant, *F*(1, 31) = 0.75, *p* > 0.05. The main effect of audio-visual congruence was not significant, *F*(1, 31) = 0.09, *p* > 0.05. The main effect of presentation durations was also not significant, *F*(2,62) = 2.88, *p* = 0.064, *η*^*2*^ = 0.09.

**Fig 6:**
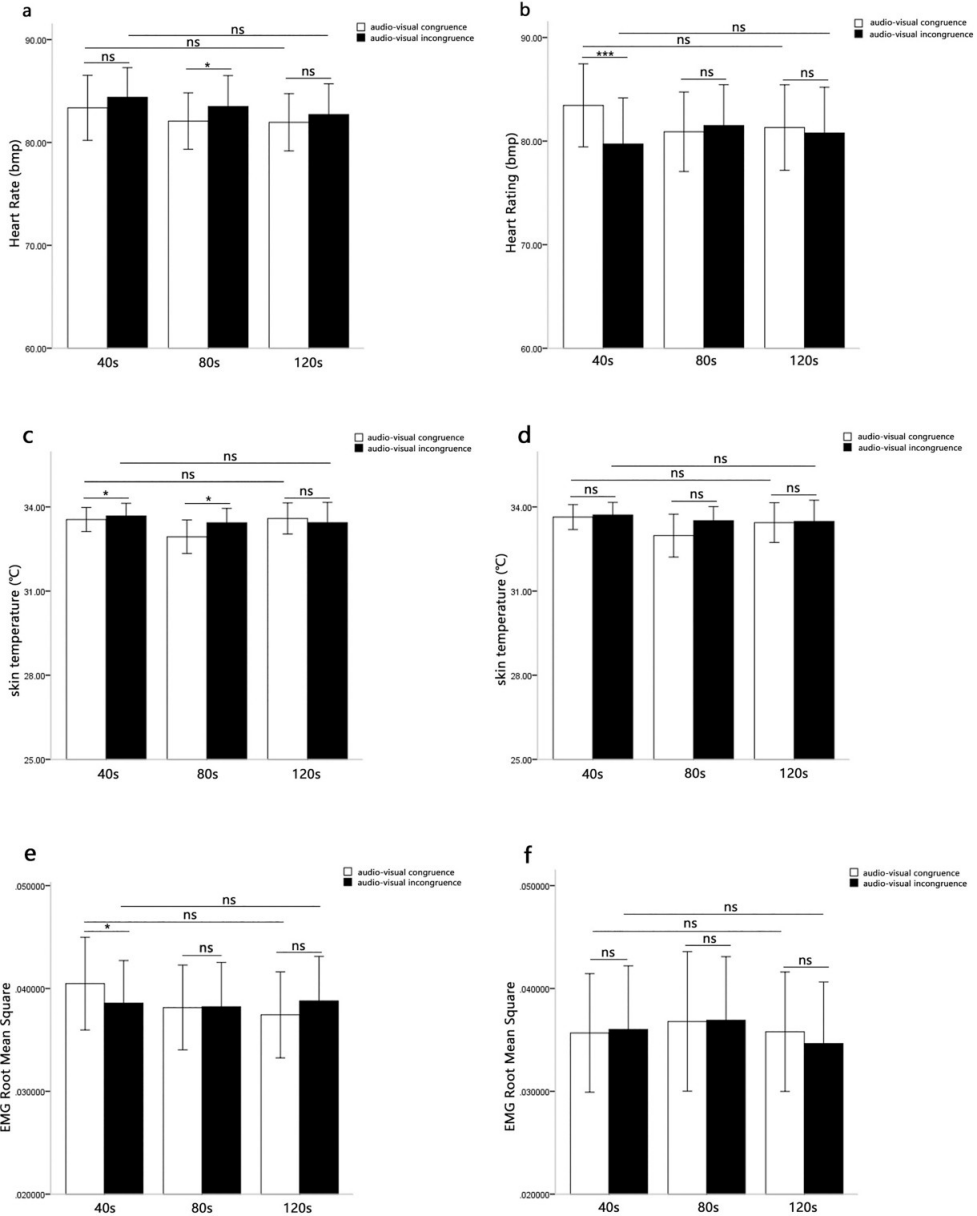
Means physiological indicators of music-induced emotion as a function of audio-visual congruence and presentation durations. Fig 6A displays the results of Repeated measures analysis in heart rates when the auditory stimuli (music) were positive. Fig 6B displays the results of Repeated measures analysis in heart rates when the auditory stimuli (music) were negative. Fig 6C displays the results of Repeated measures analysis in skin temperatures when the auditory stimuli (music) were positive. Fig 6D displays the results of Repeated measures analysis in skin temperatures when the auditory stimuli (music) were negative. Fig 6E displays the results of Repeated measures analysis in EMG root mean squares when the auditory stimuli (music) were positive. Fig 6F displays the results of Repeated measures analysis in EMG root mean squares when the auditory stimuli (music) were negative.

However, the three-way interaction of audio-visual congruence, music type and presentation durations was not significant, *F*(2, 62) = 2.36, *p* > 0.05. Furthermore, the interaction between audio-visual congruence and presentation durations was not significant for the positive music, *F*(2, 62) = 0.21, *p* > 0.05 (Fig 6A). The interaction between audio-visual congruence and presentation durations was significant for the negative music, *F*(2, 62) = 7.14, *p* < 0.01, *η*^*2*^ = 0.19. Simple effect analysis showed the audio-visual congruence effects were only significant at 40s, *F*(2, 62) = 21.52, *p* < 0.001, *η*^*2*^ = 0.41, and the heart rates was higher for audio-visual congruence than for audio-visual incongruence. The audio-visual congruence effects were not significant at 80s, *F*(2, 62) = 0.49, *p* > 0.05, or at 120s, *F*(2, 62) = 0.34, *p* > 0.05 (Fig 6B).

#### Skin temperature

A 2 (music type: positive emotion, negative emotion) × 2 (audio-visual congruence: audio-visual congruent, audio-visual incongruent) × 3 (presentation durations: 40s, 80s, 120s) repeated-measures ANOVA was performed on the skin temperature. The main effect of audio-visual congruence was significant, *F*(1, 31) = 4.54, *p* < 0.05, *η*^*2*^ = 0.13. The main effect of music type was not significant, *F*(1, 31) = 0.22, *p* > 0.05. The main effect of presentation durations was also not significant, *F*(2,62) = 2.20, *p* > 0.05.

The three-way interaction of audio-visual congruence, music type and presentation durations was not significant, *F*(2, 62) = 0.94, *p* > 0.05. Furthermore, the interaction between audio-visual congruence and presentation durations was significant for the positive music, *F*(2, 62) = 4.73, *p* < 0.05, *η*^*2*^ = 0.13. Simple effect analysis showed the audio-visual congruence effects were significant at 40s (*F*(2, 62) = 5.47, *p* < 0.05, *η*^*2*^ = 0.15) and 80s (*F*(2, 62) = 5.14, *p* < 0.05, *η*^*2*^ = 0.14), and the skin temperature was lower for audio-visual congruence than for audio-visual incongruence. The audio-visual congruence effects were not significant at 120s, *F*(2, 62) = 1.02, *p* > 0.05 (Fig 6C). The interaction between audio-visual congruence and presentation durations was not significant for the negative music, *F*(2, 62) = 2.94, *p* = 0.06, *η*^*2*^ = 0.09 (Fig 6D).

#### EMG root mean square

A 2 (music type: positive emotion, negative emotion) × 2 (audio-visual congruence: audio-visual congruent, audio-visual incongruent) × 3 (presentation durations: 40s, 80s, 120s) repeated-measures ANOVA was performed on EMG root mean square. The main effect of presentation durations was significant, *F*(2,62) = 3.24, *p* < 0.05, *η*^*2*^ = 0.09. The EMG root mean square was lower in the audio-visual incongruence than in the audio-visual congruence. The main effect of audio-visual congruence was not significant, *F*(1, 31) = 0.98, *p* > 0.05. The main effect of music type was not significant, *F*(1, 31) = 1.31, *p* > 0.05.

The three-way interaction of audio-visual congruence, music type and presentation durations was not significant, *F*(2, 62) = 2.57, *p* = 0.08, *η*^*2*^ = 0.08. Furthermore, the interaction between audio-visual congruence and presentation durations was significant for the positive music, *F*(2, 62) = 4.93, *p* < 0.05, *η*^*2*^ = 0.14. Simple effect analysis showed the audio-visual congruence effects were only significant at 40s, *F*(2, 62) = 7.55, *p* < 0.05, *η*^*2*^ = 0.20, and the EMG root mean square was higher for audio-visual congruence than for audio-visual incongruence. The audio-visual congruence effects were not significant at 80s (*F*(2, 62) = 0.05, *p* > 0.05) and 120s (*F*(2, 62) = 2.00, *p* > 0.05) (Fig 6E). The interaction between audio-visual congruence and presentation durations was not significant for the negative music, *F*(2, 62) = 0.92, *p* > 0.05 (Fig 6F).

#### Prefrontal EEG

**Fig 7:**
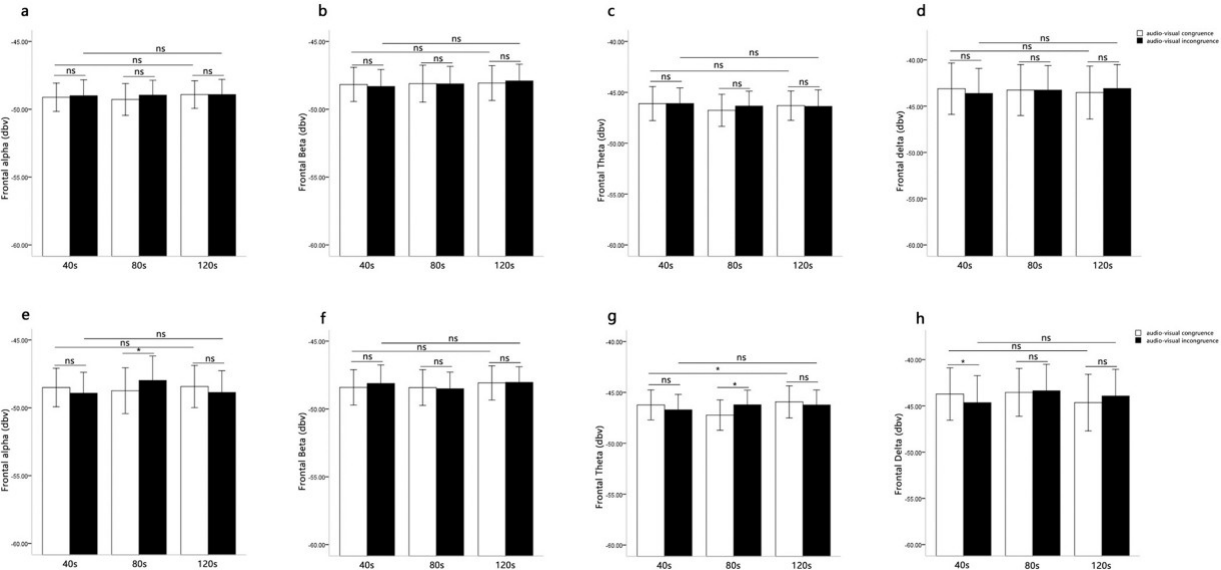
Means EEG power of music-induced emotion as a function of audio-visual congruence and presentation durations. Fig 7A–7D display the results of Repeated measures analysis when the auditory stimuli (music) were positive. Fig 7E–7H display the results of Repeated measures analysis when the auditory stimuli (music) were negative.

In the alpha power, the three-way repeated measure ANOVA revealed that the main effect of music type was not significant, *F*(1, 31) = 0.34, *p* > 0.05. The main effect of audio-visual congruence was not significant, *F*(1, 31) = 0.22, *p* > 0.05. The main effect of presentation durations was not significant, *F*(2,62) = 0.47, *p* > 0.05. The three-way interaction of audio-visual congruence, music type and presentation durations was not significant, *F*(2, 62) = 0.89, *p* > 0.05. Furthermore, the interaction between audio-visual congruence and presentation durations was not significant for the positive music, *F*(2, 62) = 0.34, *p* > 0.05 (Fig 7A). The interaction between audio-visual congruence and presentation durations was significant for the negative music, *F*(2, 62) = 3.10, *p* = 0.05, *η*^*2*^ = 0.09. Simple effect analysis showed the audio-visual congruence effects were only significant at 80s, *F*(2, 62) = 4.36, *p* < 0.05, *η*^*2*^ = 0.12, and the alpha power was lower for audio-visual congruence than for audio-visual incongruence. The audio-visual congruence effects were not significant at 40s (*F*(2, 62) = 1.57, *p* > 0.05) and 120s (*F*(2, 62) = 1.01, *p* > 0.05) (Fig 7E).

In the beta power, the three-way repeated measure ANOVA revealed that the main effect of presentation durations was significant, *F*(2, 62) = 3.49, *p* < 0.05, *η*^*2*^ = 0.10. The beta power was higher in the audio-visual incongruence than in the audio-visual congruence and control group. The main effect of music type was not significant, *F*(1,31) = 1.64, *p* > 0.05. The main effect of audio-visual congruence was not significant, *F*(1, 31) = 0.27, *p* > 0.05. The three-way interaction of audio-visual congruence, music type and presentation durations was not significant, *F*(2, 62) = 1.09, *p* > 0.05. Furthermore, the interaction between audio-visual congruence and presentation durations was not significant for the positive music, *F*(2, 62) = 0.54, *p* > 0.05 (Fig 7B). The interaction between audio-visual congruence and presentation durations was not significant for the negative music, *F*(2, 62) = 0.58, *p* > 0.05 (Fig 7F).

In the theta power, the three-way repeated measure ANOVA revealed that the main effect of music type was not significant, *F*(1, 31) = 0.20, *p* > 0.05. The main effect of audio-visual congruence was not significant, *F*(1, 31) = 0.45, *p* > 0.05. The main effect of presentation durations was not significant, *F*(2,62) = 0.20, *p* > 0.05. The three-way interaction of audio-visual congruence, music type and presentation durations was not significant, *F*(2, 62) = 1.35, *p* > 0.05. Furthermore, the interaction between audio-visual congruence and presentation durations was not significant for the positive music, *F*(2, 62) = 0.57, *p* > 0.05 (Fig 7C). The interaction between audio-visual congruence and presentation durations was significant for the negative music, *F*(2, 62) = 4.41, *p* < 0.05, *η*^*2*^ = 0.09. Simple effect analysis showed the audio-visual congruence effects were only significant at 80s, *F*(2, 62) = 6.53, *p* < 0.05, *η*^*2*^ = 0.17, and the theta power was higher for audio-visual congruence than for audio-visual incongruence. The audio-visual congruence effects were not significant at 40s (*F*(2, 62) = 1.82, *p* > 0.05) and 120s (*F*(2, 62) = 0.84, *p* > 0.05) (Fig 7G).

In the delta power, the three-way repeated measure ANOVA revealed that the main effect of music type was not significant, *F*(1, 31) = 0.42, *p* > 0.05. The main effect of audio-visual congruence was not significant, *F*(1, 31) = 0.02, *p* > 0.05. The main effect of presentation durations was not significant, *F*(2,62) = 1.66, *p* > 0.05. The three-way interaction of audio-visual congruence, music type and presentation durations was not significant, *F*(2, 62) = 0.35, *p* > 0.05. Furthermore, the interaction between audio-visual congruence and presentation durations was not significant for the positive music, *F*(2, 62) = 1.43, *p* > 0.05 (Fig 7D). The interaction between audio-visual congruence and presentation durations was significant for the negative music, *F*(2, 62) = 3.28, *p* < 0.05, *η*^*2*^ = 0.10. Simple effect analysis showed the audio-visual congruence effects were only significant at 40s, *F*(2, 62) = 4.65, *p* < 0.05, *η*^*2*^ = 0.13, and the delta power was higher for audio-visual congruence than for audio-visual incongruence. The audio-visual congruence effects were not significant at 80s (*F*(2, 62) = 0.18, *p* > 0.05) and 120s (*F*(2, 62) = 2.03, *p* > 0.05) (Fig 7H).

### Discussion

In Experiment 2, the present study sought to explore the modulation effect of presentation durations of visual information on music-induced emotion. On the one hand, the results demonstrated the same conclusion as in Experiment 1: that the participants appeared to combine both sets of sensory (i.e., visual and auditory) information when presented with the face and music synchronously. On the other hand, the results revealed that the integration effect was strongest when the visual information presentation durations were within 80s. Moreover, for the negative music, the presentation durations of visual information would modulate the integration effect in the indexes of heart rates, alpha power, theta power and delta power of prefrontal area. However, for the positive music, the presentation durations did not have significant influence in the music-induced emotion. Specifically, the differences of audio-visual congruence were significant both at 40s and 80s conditions.

## General discussion

The present study used combined behavioral/physiological measures to examine the audio-visual integration on music-induced emotion. Three main findings emerged: First, in line with previous findings as well as with our own hypothesis, the results showed that, relative to the audio-only presentation, the audio-visual congruent presentation led to a more intense emotional response. Second, the audio-visual integration occurred in both the positive music and the negative music. Meanwhile, the audio-visual integration effect was larger for positive music than for negative music. Finally, when the visual information presentation durations were within 80s, visual stimuli has a significant influence on the music-induced emotion when the music is negative, which indicated that the integration effect was more likely to occur in the negative music.

Based on previous findings, the present study explored whether audio-visual integration would occur on the music-induced emotion processing. According to previous studies, the multisensory integration had two main types of behavioral outcomes: the multisensory illusion effects (e.g., the McGurk effect, the double-flash illusion, and the ventriloquism effect) and multisensory performance improvement effects (e.g., the redundant signal effect) [1]. The redundant signal effect suggests that when stimuli from multiple sensory modalities are presented synchronously, the subjective responses can be faster and more accurate than when the same stimuli are presented from a single sensory modality [8, 13, 32]. The present study also showed that the audio-visual congruent presentation led to a more intense emotional response than the audio-only presentation. Specifically, relative to the audio-only presentation, the audio-visual congruent presentation led to a higher self-reported intensity of experienced emotion, EMG root mean square, alpha power, beta power, and lower heart rate. These results were in line with some previous studies [23, 26], which also found that visual information had a stronger effect on the music-induced emotion. For example, Chapados and Levitin reported that, compared to audio-only presentation, audio-visual congruent presentation induced a higher response in skin conductance [23]. Jeong et al. observed that the congruence of musical emotion and facial emotion resulted in the increased activation in STG and the decreased activation in FG [28]. Studies that utilized the Frontal area, e.g., Medial-frontal, Fronto-central and Fronto-parietal, reported an occurrence of the audio-visual integration effect [33–36]. However, some results differed by showing that the audio-visual congruent presentation did not result in a more intense emotional response [25]. These inconsistent results were directional to the different modulation of multisensory processing by selective-modality and divided-modality attention [1]. One possible explanation for this difference was that the different experiment parameters and tasks might have contributed to the different neural changes and behavioral responses. Nevertheless, these results confirmed the importance of visual information in the audio-visual integration [20, 37] and that audio-visual integration occurred in musical emotion.

The findings in the audio-visual incongruent condition were in line with previous reports that the audio-visual incongruent presentation led to the participants’ decreased emotional response in the theta power and delta power of prefrontal areas. In other words, an interference effect was found on the music-induced emotion when the auditory and visual emotional information were incongruent. First, this was in line with previous studies on emotional conflicts. The present study indicated that there was an emotional conflict effect because of the presence of different visual emotional cues [38]. Second, this result suggested that individuals would integrate visual and auditory information meaningfully when visual and auditory information were incongruent [8, 39]. Visual emotional information played a greater role in the audio-visual integration [20, 37], but bimodal emotional cues could integrate into a coherent percept during emotional change perception regardless of attention direction [22]. Thus, even though there was a strong interference on the music-induced emotion, the participant still elicited the same emotion as the auditory information. This could be due to the phenomenon that when an object-related congruency exists between the positive visual information and the negative auditory information, the representation-driven spread attention will occur [1]. Previous studies found that the incongruent condition resulted in a stronger distraction than the congruent condition and that the incongruent condition obtained more intensive attention [40]. Based on this fact, when the information had a conflict, the attention would spread. Thus, participants were more aware of visual information, causing the interference effect to occur.

Additionally, the present study’s results showed that the difference among the three conditions (audio-visual congruence, audio-visual incongruence and audio only) was larger for the emotional response elicited by the positive music than by the negative music. Specifically, relative to the audio-visual incongruent condition, the audio-visual congruent presentation led to a higher self-reported intensity of experienced emotion, EMG root mean square, alpha power, and beta power of the prefrontal area for the positive music than for the negative music. However, for the other indexes (i.e., heart rate, theta power, and delta power), the opposite results occurred: namely, that the difference between the audio-visual congruent condition and the audio-visual incongruent condition was greater for the negative music than for the positive music in the music-induced emotion. There are two possible explanations for these results. First, according to the attention distribution theory, negative emotions can narrow one’s breadth of attention, while positive emotions tend to broaden one’s breadth of attention [30]. In the present study, the positive music broadened the range of attention [31] and participants were more likely to be attracted by the visual information. Therefore, when the picture emotion and music emotion were both positive types, the individual was able to perceive the emotion of the picture and integrate it more easily. However, when the valence of the picture was negative, the visual emotional information played a greater role in the audio-visual integration [20, 37]. Thus, the individual’s positive emotional experience was more likely to be affected by the picture, resulting in greater differences between congruence and incongruence. In contrast, when the music was negative, the range of individuals’ attention was smaller, so they needed more time and attention to feel the musical emotion instead of the picture. Thus, the picture had little effect on the individuals, and the difference between the congruent condition and the incongruent condition decreased. Second, there were different neural bases for the integration of different emotion [12, 41]. For example, happy audio-visual pairs of activating structures were mainly lateralized in the left hemisphere, whereas fearful audio-visual pairs of activating structures were lateralized in the right hemisphere [12]. In other words, due to the different neural bases for the different emotions, the audio-visual integration effect was different.

Furthermore, presentation durations of visual information had a greater modulation effect in the audio-visual integration. Specifically, when the visual information presentation durations were within 80s, visual stimuli has a significant influence on the music-induced emotion. Moreover, the modulation effect of presentation durations of visual information was significant for the negative music reflected the indexes of heart rates, alpha power, theta power and delta power of prefrontal area. However, the modulation effect of presentation durations of visual information was not significant for the positive music. Specifically, the differences of audio-visual congruence were significant both at 40s and 80s conditions. These results were in line with our hypothesis and with previous findings. Specifically, when the two sensory modalities were presented at the same time, or the time window was at 300ms, the information from the two sensory modalities would be integrated [15, 16, 42]. In the present study, when the visual information (i.e., facial pictures) and the auditory information (i.e., music) were presented synchronously, the audio-visual integration effect was observed in all presentation duration conditions. These results were also consistent with the previous study on audio-visual emotional integration in which S-mSTC (Synchrony-defined multisensory superior temporal cortex) responded only when auditory and visual stimuli were synchronous [18]. Therefore, the present study indicated that the audio-visual integration effect occurred only when the visual and auditory information were synchronous.

In addition, we found some special results in negative music. In experiment 1, we found that, relative to the audio-only presentation, the audio-visual congruent presentation led to a more intense emotional response. However, when auditory stimuli and visual stimuli were different, the visual didn’t influence the music-induced emotion. In experiment 2, we found that presentation durations of visual information had a greater modulation effect in the audio-visual integration. Specifically, when the visual information presentation durations were within 80s, visual stimuli has a significant influence on the music-induced emotion. The possible explanations for these results were that the time course of audio-visual integration brought out the effect. In the early stage of music playing, visual stimuli had a significant effect on the music-induced emotion. When visual and auditory information were congruent, visual emotion could increase the individual's auditory experience. When the visual and auditory information were incongruent, the visual information would interfere with the emotional induction of music, resulting in greater differences between congruence and incongruence. Correspondingly, when the presentation durations of music reached a certain time, the audio-visual integration was completed, and the difference between audio-visual congruence and incongruence was small. Therefore, we did not find significant differences between the audio-visual congruence and the audio-visual incongruence at 120s. According to the previous study, the combination between visual and auditory sensory modalities occurred at an early stage. For example, Brett-Green et al. found that significant audio-visual integration effect occurred in central/post-central scalp regions between 180-220ms in both hemispheres as well as midline scalp regions [43]. Several studies had shown that 1s was enough for music to induce a specific emotion [44–46]. However, more studies have shown that the music should be last for a relatively long time to induce a specific emotion in well. For example, Baumgartner studied the combination of the music and pictures, and the music presentation time was 70 seconds [44, 45]. Chapados et al and Vuoskoski et al investigated whether the visual stimuli effected the music-induced emotion, which their music presentation time was about 3 minutes [23, 25]. Correspondly, these indicated that the integration between the audio and visual madality needed long durations in the music-induced emotion.

In addition to valence (pleasantness), the primary dimensions of emotion include arousal, familiarity, etc. Marin et al applied a crossmodal priming paradigm to investigate the role of arousal in crossmodal emotional priming between the musical and visual domain by focusing on felt emotions. They found that arousal played a crucial role in emotional processing and music-induced emotions were significantly modulated by the arousal but not the pleasantness [47]. However, many researches suggested that valence played an important role in audio-visual integration. For example, the structures activated by happy audio-visual pairs were mainly lateralized in the left hemisphere, whereas the structures activated by fearful audio-visual pairs were lateralized in the right hemisphere [12]. Park et al. found that different emotions had different emotional processing mechanisms, such as fearful audio-visual integration processing in the posterior cingulate gyrus, spindle gyrus and cerebellum, while happy audio-visual integration processing in the middle temporal gyrus, hippocampus [11]. In the present study, the dimension of arousal was controlled and we just focused on the effect of audio-visual integration modulated by the pleasantness. Whether the pleasantness and arousal impacted the audio-visual integration together or separately, it should be further explored to obtain more evidence. Moreover, compared with the traditional S-R (Stimuli-Response) experiments, the number of stimuli in present study is a little bit small. We could take more stimuli and more trials to get more data and increase the stability of the experiment in a future study.

In conclusion, the present study suggested that the audio-visual integration effect (i.e., the redundant signals effect) also existed in the music-induced emotion. The present study revealed that emotion could be enhanced when the audio and visual emotions were consistent. Moreover, when the music was positive, the effect of audio-visual integration was more significant. When the music was negative, the modulation effect of the presentation durations of visual information on the music-induced emotion was more significant.

## Supporting information

S1 Dataset Experiment 1 data. (XLSX)

S2 Dataset Experiment 2 data. (XLSX)
